# Women in Chemistry: Q&A with Professor Malika Jeffries-EL

**DOI:** 10.1038/s42004-024-01314-z

**Published:** 2024-10-11

**Keywords:** Semiconductors, Electronic materials

## Abstract

Malika Jeffries-EL is the Senior Dean of the Graduate School in Arts and Sciences and Professor of Chemistry and Materials Science at Boston University. Professor Jeffries-EL’s research focuses on the development of organic semiconductors—materials that combine the processing properties of polymers with the electronic properties of semiconductors.

Malika Jeffries-EL has authored over 50 peer-reviewed publications and has given over 200 lectures domestically and abroad. She is a Fellow of the American Chemical Society (ACS), fellow of the Royal Society of Chemistry and has won numerous awards including the ACS Stanley C. Israel Regional Award for Advancing Diversity in the Chemical Sciences. She is currently an Associate Editor for Chemical Science and has also served on the editorial boards for the Journal of Materials Chemistry C and Materials Advances, and the editorial advisory boards for ACS Central Science, and Chemical and Engineering News. Professor Jeffries-EL is a staunch advocate for diversity and dedicated volunteer that has served in several activities within the ACS and currently serves as an elected member of the board of directors, as a director-at-large. She is also a science communicator who seeks to encourage students from underrepresented groups to pursue STEM degrees and recently appeared on NOVA series Beyond the Elements. She also serves the community through her work with Alpha Kappa Alpha Sorority, Incorporated. Dr Jeffries-EL is a native of Brooklyn, New York.

**Figure:**
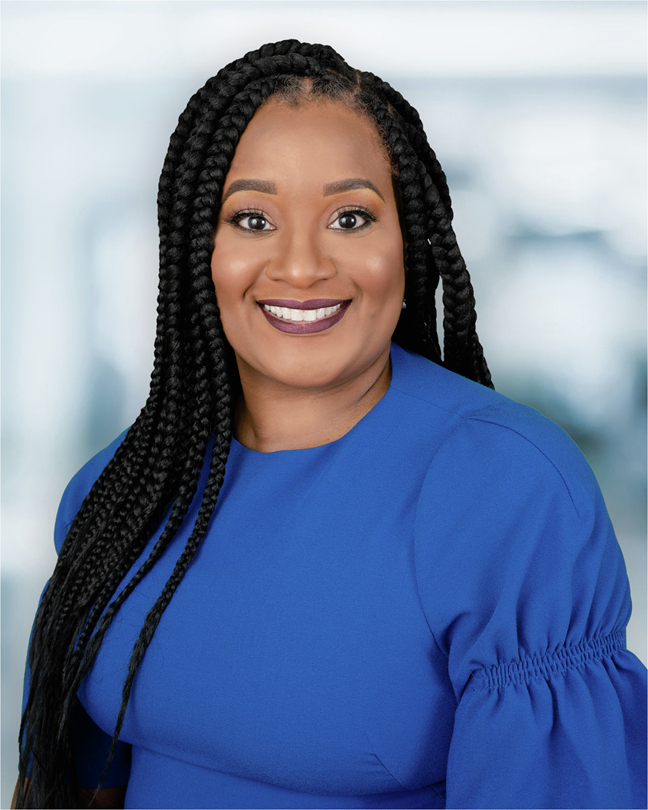
Malika Jeffries-EL

Why did you choose to be a scientist?

I was always curious about the workings of the world: how water transformed into ice when chilled and steam when heated. And why did baking soda and vinegar produce a foamy reaction? How do caterpillars undergo metamorphosis to become butterflies?

What scientific development are you currently most excited about?

I am thrilled about the comeback of organic light-emitting diodes. The effectiveness of traditional OLEDs is restricted by the fact that only 25% of the produced excitons are singlets. However, scientists are now using thermally activated delayed fluorescence (TADF) to transform the non-emitting triplet excitons into emitting singlet excitons through reverse intersystem crossing. Consequently, the internal quantum efficiency of OLEDs based on TADF can reach up to 100%. Additional enhancements can be achieved through hyperfluorescence, which operates by transferring energy from triplet excitons to TADF molecules, which then convert the energy to singlet excitons that can be re-emitted by fluorescent molecules, improving the overall efficiency of light production. This line of research will result in more efficient OLEDs for various applications.

What direction do you think your research field should go in?

We must harness the power of AI to accelerate materials discovery and develop new models that can more accurately predict material properties. A key part of this effort involves expanding the size of training datasets to improve the accuracy and reliability of predictions. However, a significant challenge arises from the prevalent bias in available data, as researchers tend to publish only positive results. This practice skews models and limits their learning potential. We must foster a culture where scientists share both positive and negative results. Failures can provide valuable insights, and including this data can enhance the model and lead to more accurate predictions of material properties.

What do you most (and least) enjoy about being a scientific researcher?

I enjoy the thrill of discovery, whether it’s the excitement of interpreting a NMR spectra for a new molecule or finding that a material you’ve designed meets or exceeds expectations. I am not too fond of failed reactions and experiments, but I know that is the price to pay for forging new pathways.

How can individual scientists support and celebrate their women colleagues?

Promote them in spaces they haven’t been invited into. Brag about their work, and recommend them for seminars and conferences. Nominate them for awards.

How can publishers, editors, funders and conference organizers better support women scientists?

This group has tremendous power and is well-positioned to help advance women scientists by being aware of bias and working to resolve it. Provide bias training to anyone who works as a reviewer or organizer. Flag people in systems who submit reviews that indicate evidence of bias so they are not assigned to review work by women. Call out organizers who fail to invite women to speak at their meetings, even boycott them.

Do you have any advice you would like to share with women starting out in chemical research?

Create a village to support you throughout your career. This includes friends and family who can support you emotionally, along with mentors, sponsors and coaches who can help you advance professionally. The best mentoring relationships are those that form organically, so take the time to network with senior people in your field. Professional societies are excellent venues to facilitate this.

Are you or have you been supported by a mentor?

I have been fortunate to have had several excellent mentors throughout my career, both male and female. Each one brings a different perspective and I have benefited greatly from them all.

What was the best advice you received?

If you don’t make time for your wellness, you will take time for your illness, from my grandma Jean. Who reminded me that no career is more important than your health.

*This interview was conducted by the editors of Communications Chemistry*.

